# Implementing solutions to improve and expand telehealth adoption: participatory action research in four community healthcare settings

**DOI:** 10.1186/s12913-015-1195-3

**Published:** 2015-12-01

**Authors:** Johanna Taylor, Elizabeth Coates, Bridgette Wessels, Gail Mountain, Mark S. Hawley

**Affiliations:** Department of Health Sciences, University of York, Area 4, 2nd Floor, ARRC Building, Heslington, York YO10 5DD UK; School of Health and Related Research, University of Sheffield, Regent Court, 30 Regent Street, Sheffield, S1 4DA UK; Department of Sociological Studies, University of Sheffield, Elmfield, Northumberland Road, Sheffield, S10 2TU UK

**Keywords:** Technology, Telehealth, Adoption, Implementation, Action research, PDSA, Community nursing, Service improvement, Long-term conditions

## Abstract

**Background:**

Adoption of telehealth has been slower than anticipated, and little is known about the service improvements that help to embed telehealth into routine practice or the role of frontline staff in improving adoption. This paper reports on participatory action research carried out in four community health settings using telehealth for patients with Chronic Obstructive Pulmonary Disease and Chronic Heart Failure.

**Methods:**

To inform the action research, in-depth case studies of each telehealth service were conducted (May 2012–June 2013). Each service was then supported by researchers through two cycles of action research to implement changes to increase adoption of telehealth, completed over a seven month period (July 2013–April 2014). The action research was studied via observation of multi-stakeholder workshops, analysis of implementation plans, and focus groups.

**Results:**

Action research participants included 57 staff and one patient, with between eight and 20 participants per site. The case study findings were identified as a key source of information for planning change, with sites addressing common challenges identified through this work. For example, refining referral criteria; standardizing how and when patients are monitored; improving data sharing; and establishing evaluation processes. Sites also focused on raising awareness of telehealth to increase adoption in other clinical teams and to help secure future financial investment for telehealth, which was required because of short-term funding arrangements. Specific solutions varied due to local infrastructures, resources, and opinion, as well as previous service developments. Local telehealth champions played an important role in engaging multiple stakeholders in the study.

**Conclusions:**

Action research enabled services to make planned changes to telehealth and share learning across multiple stakeholders about how and when to use telehealth. However, adoption was impeded by continual changes affecting telehealth and wider service provision, which also hindered implementation efforts and affected motivation of staff to engage with the action research, particularly where local decision-makers were not engaged in the study. Wider technological barriers also limited the potential for change, as did uncertainties about goals for telehealth investment, thereby making it difficult to identify outcomes for demonstrating the added value over existing practice.

## Background

Implementation of telehealth, defined as technologies that enable patients and clinicians to exchange information about their health state remotely [1], has suffered from slow adoption and ‘pilotitis’ in parts of the UK and Europe [2, 3]. Reasons for the failure of telehealth to become part of routine care are multi-faceted, with the mixed evidence of cost effectiveness often cited as one of the leading factors [4–6]. More specifically, inconclusive results from the Whole Systems Demonstrator trial in England are often referenced in discussions about the inability of telehealth to be scaled up in the UK [7–9]. One of the challenges to evaluating the effects of telehealth relates to the limited knowledge of how factors affecting both adoption, which we define as the ‘processes involved in making the decision to use the technology…located at different levels (individual, organizational, regional, national)’ ([10]: p96), and implementation, which we refer to as the ‘active and planned efforts to mainstream an innovation within an organisation’ ([11]: p582), may influence the results [1]. However, little research has examined the on-going processes of implementing telehealth or focused on how challenges can be overcome to increase adoption and embed telehealth into routine care [2, 12, 13].

More recent studies have started to identify barriers affecting implementation, which include limitations of telehealth equipment, technological and organizational barriers to sharing patient data and working across service boundaries, low staff awareness and engagement, increased staff workload, inefficient resources to support service delivery, difficulties assessing patient suitability for telehealth, and uncertainty about the structures and processes that are required to monitor patients remotely [14–17]. Whilst certain barriers affecting adoption are specific to telehealth, other identified factors are evident in research examining adoption of new innovations more generally [18]. For example, fear of increased workload and concerns about job roles and patient care, not involving frontline staff in planning and service design, poor staff training, and conversely, the importance of frontline champions who can help to increase awareness and spread success [11].

For telehealth, the range of devices and applications that are now available, and the multiple stakeholders and mechanisms involved in any single service adds further complexity to implementation, and consequently to the challenges of normalizing into healthcare delivery [4]. Each implementation of telehealth is both unique to the specific clinical setting and patient population, but also to the service design and specification [6]. Local configuration and management of health and social care provision also shape deployment and implementation of new technologies [1]. Moreover, because there are competing visions and goals for telehealth [19], there are resulting mixed opinions about what it should and can achieve [15]. This highlights the social complexities surrounding both the intervention and the system into which it is introduced [20], and raises additional questions about evaluation when what we are deploying is ambiguously defined.

Examining the active and planned efforts to implement telehealth in different healthcare settings, and identifying solutions to address identified barriers to normalizing telehealth, is therefore essential if we are to inform the development of local services that can avoid current pitfalls and problems, and to help design future studies that can better examine effectiveness. A recognized challenge of moving from research to practice is that pilots and trials are often time-limited, with little consideration to what will happen beyond completion [3]. Current research has focused more upon measuring clinical outcomes and describing patient experience within the context of randomised controlled trials [7–9, 12], but few studies have examined the implementation of telehealth within routine care, or the efforts to use telehealth over an extended time period. This paper reports the findings of a two-phase action research study, which aimed to identify the factors affecting telehealth adoption, and test solutions to address prioritized areas for improvement and expansion.

## Methods

### Study design

In-depth qualitative case studies were conducted to inform the development of a programme of participatory action research to improve adoption of telehealth, carried out using principles of the Plan Do Study Act (PDSA) service improvement tool [21]. The research took place within four community nursing settings using telehealth to monitor the symptoms of patients with Chronic Obstructive Pulmonary Disease (COPD) and Chronic Heart Failure (CHF) who were living at home. These conditions were selected for the study because they are two of the most common long-term conditions for which telehealth is currently used [6].

It is important to note that, given the emergent nature of the evidence base regarding clinical and cost-effectiveness of telehealth, the researchers approached the action research from a neutral position on the issue of whether telehealth should be expanded. The researchers recognise the implicit complexity of maintaining a neutral standpoint, particularly as the research rationale set by the funders was to identify solutions to help mainstream telehealth. To manage this complexity, and in keeping with the principles of participatory action research, participants in each site were encouraged to draw upon the case study findings and their own local knowledge of their telehealth service to select actions to test as part of the study, and to define and assess improvement as part of this process.

### Study sites

Each setting included community matron and specialist nursing teams who case manage patients with COPD and CHF, and who were using telehealth to monitor patients’ vital signs and symptoms. The sites varied in size and organizational structure, and caseloads held by different teams. The sites also differed in terms of when telehealth had been introduced, the number of units available and in use, and the stakeholders involved in management and delivery (see Table 1).

**Table 1:** Study site characteristics

Research site identifier	Site A	Site B	Site C	Site D
Telehealth deployment				
Year of introduction	2007	2009	2006	2010
Equipment purchased (P) or leased (L)	P	P	L	P
No. of telehealth units available for use^a^	165	79	n/a	40
No. of telehealth units in use^b^	104	39	200	34
Referral routes into telehealth				
Community matrons	✓	✓	✓	✓
Case managers	✓			✓
Specialist respiratory nurses	✓	✓	✓	
Specialist heart failure nurses	✓	✓	✓	
GPs	✓			
Telehealth stakeholders				
NHS community healthcare provider	✓		✓	✓
NHS hospital trust	✓			✓
Clinical commissioning group		✓	✓	
Equipment manufacturer	✓	✓	✓	✓
Local authority	✓	✓	✓	
Community interest company		✓	✓	
Private company		✓	✓	

^a^Estimates from case study data

^b^At end of data collection period in phase one

The telehealth equipment provided to patients to monitor their symptoms varied by functionality and complexity, and had been purchased or leased by different technology suppliers in each site. One site used smart phone and mobile broadband technology; others used specialist telehealth units and either wireless or wired connection in patients’ homes. All units utilized vital sign peripherals (e.g. blood pressure cuffs, weighing scales) and asked questions on screen about a patient’s health and symptoms. Units automatically uploaded patient data to a secure online platform owned by the technology provider, which compared patients’ readings against individual parameters and generated alerts for patients whose symptoms indicated a potential problem.

All sites checked the system daily for alerts, and had developed a triage system to make sure that patients who required clinical input would be contacted by their community nurse. The daily monitoring and first line triage of patients using telehealth varied across the sites and over time as the services developed, using registered nurses, clinical support workers, administrative staff, or outsourcing the role to a telehealth company or local authority.

### Ethical approval

The South West–Frenchay National Research Ethics Committee in the UK granted ethical approval for the study (reference 13/SW/0036). Access to NHS sites was granted via local NHS research offices, and for participating local authorities, community interest groups and private companies via internal governance procedures.

### Phase one – case studies

In-depth case studies were carried out to examine referral and care pathways for telehealth; explore usage and acceptance among staff and patients; and to identify factors affecting telehealth adoption. Detailed findings from this work are reported elsewhere [15]. The case studies also helped to identify the main stakeholder groups involved in telehealth and were a resource to inform the development of solutions to improve telehealth in phase two.

Purposive sampling strategies were used to gather views from different stakeholders, who were defined as ‘an individual or organisation that can affect or is affected by [telehealth]’ ([22]: p751). Across the sites, a total of 105 staff and 40 patients (with 12 carers contributing) were interviewed between May 2012 and June 2013 (see Table 2). The study employed a semi-structured interview guide to cover topics identified from an earlier systematic review of factors affecting staff adoption of telehealth [12]. Other documentary sources were also analyzed, for example referral guidelines, local evaluations of telehealth and standard operating procedures. Thematic analysis of data utilized the Framework approach [23], and primarily focused on identifying factors affecting telehealth adoption.

**Table 2:** Case study participant types

Participant type	Site A	Site B	Site C	Site D	Total
Registered community nurses^a^	15	13	12	18	58
Other frontline staff^b^	4	4	3	5	16
Clinical leads and service managers	2	0	6	2	10
Other managers	6	6	7	2	21
Patients	9	6	12	13	40
Carers	1	1	4	6	12
Total	37	30	44	46	157

^a^Included community matrons, specialist nurses, case managers, district nurses

^b^Included GPs, clinical support workers, telehealth installers, call handlers, administrators, technical staff

### Phase two – participatory action research

The second phase comprised a seven month programme of action research in each study site, conceptualized as ‘the study of a social situation carried out by those involved in that situation in order to improve both their practice and the quality of their understanding’ ([24]: p8). Two researchers worked in partnership with participants in each site as they planned, tested and evaluated solutions that they identified and agreed would help to improve telehealth adoption. The PDSA approach was employed to structure the action research around making improvements to their existing telehealth service, while still facilitating stakeholder engagement and shared learning [21].

One of the central features of PDSA is that more than one cycle takes place [25]. In this study, two three-month cycles of PDSA and a series of multi-stakeholder action research workshops were planned in each site, which created opportunities for shared reflection and action; a principle that is central to action research [26]. The workshops were held at the beginning of the action research (Month 1), at the mid-point following the first cycle (Month 4), and at the end following the second cycle (Month 7).

The main purpose of the initial workshop was to develop an implementation plan (PLAN). To facilitate this, participants were presented with findings from the case studies after which a whole group discussion was convened to identify, agree, and plan activities for the action research. To ensure that the work would be feasible, sites were asked to select approximately three to six actions. For each action, an Action Inquiry Group (AIG) was established, with members completing a detailed implementation plan of how the work would be carried out and evaluated, including identifying outcomes and data that could be collected for evaluation. AIG leads were given responsibility to take forward the plan during the subsequent three month cycle (DO), and review progress and summarize any learning in order that the action research could be evaluated (STUDY).

The purpose of the second workshop was to review and reflect on the work carried out, and either extend, discontinue or modify plans for the next cycle (ACT). In the final workshop, participants were asked to review the success of the action research and assess the feasibility of implementing changes that had been piloted, which is a central component of PDSA and reflects the iterative nature of service improvement methodology [27].

### Action research participants

To recruit participants, the researchers worked with site collaborators and local telehealth champions during the transition from phase one, in part to maintain engagement and also to maximize opportunities for multi-stakeholder involvement in phase two. All case study participants who expressed an interest to take part in phase two, and other individuals identified by the site collaborators as key stakeholders, were invited to take part by attending the first workshop in which information was provided and written consent obtained. Table 3 shows that 57 staff and one patient agreed to take part in the action research, with between eight and 20 participants in each site. Staff members included commissioners, service managers, nursing and other frontline staff.

**Table 3:** Action research participant types

Participant type	Site A	Site B	Site C	Site D	Total
Community matrons and nurse specialists	8	4	5	4	21
Other frontline clinical and support staff	1	1	7	7	16
Clinical leads and service managers	5	1	3	5	14
Other managers	0	1	5	0	6
Patients	0	1	0	0	1
Carers	0	0	0	0	0
Total	14	8	20	16	58

Although patients and their carers were not excluded from phase two of the research, only one of the AIGs in site B included a patient representative, who was only able to attend the initial workshop. The implementation plans for all sites focused upon service design and delivery issues, and given that patient acceptance was not perceived as a major barrier to adoption in the participating services [15], the site collaborators decided that it was not ethically appropriate to explicitly include patients and carers. Many of the issues to be addressed in phase two highlighted the uncertainty of and problems within the telehealth services, and collaborators were concerned how this would affect patients who relied on telehealth as part of the care they received.

### Data collection and analysis

The action research was completed between July 2013 and April 2014. To examine both the process and outcomes of the action research, the study employed a combination of observation, primary data collection, and participant and researcher reflection. Two researchers worked together throughout the study period, working closely with AIG leads to enhance opportunities for reflection and interpretation. Reflective discussion during each workshop and during regular de-briefing sessions for the researchers formed part of the analytical process. Field notes from workshops and communication with participants outside of the workshops also formed a central element of the observation work, and implementation plans were updated to review progress of individual actions.

During the final workshops at each site, a focus group was conducted to discuss how successful the action research had been in taking forward service improvements, and to identify other changes affecting telehealth implementation during the same timeframe. Separate consent for the focus groups was taken at the beginning of the workshop, and a topic guide was used to structure group discussions. In total, 28 people participated in the focus groups, which each included between four and nine participants (see Table 4). Focus groups, which lasted approximately 90 minutes, were audio-recorded and transcribed verbatim. Data were analyzed thematically, which involved extracting key themes from across the data and examining relationships between themes to identify enduring barriers to normalizing telehealth into routine care.

**Table 4:** Action research participants per workshop

Number of participants attending	Site A	Site B	Site C	Site D	Total
Workshop One	8	6	10	14	38
Workshop Two	9	5	13	7	34
Workshop Three (focus groups)	6	4	9	9	28
Total participants per site	14	8	20	16	58

### Research quality

The research used a standardized approach to data collection in phase one and phase two, and an audit of processes was kept to create dependability [28]. Respondent validation was the key mechanism through which credibility of the research was assured, with key findings of each phase shared with all participants, and at multi-stakeholder workshops in each site where they were corroborated by those attending. In phase two, reflexivity within the research team, and between the researchers and action research participants, facilitated a critical and open approach to data analysis and interpretation, which is an important marker of quality in action research [29]. The final focus group conducted with phase two participants has provided further assurance of the authenticity of the findings generated from this research.

## Results

All sites identified five or six actions that would be feasible and were agreed as important to work on during the action research window. Individual AIGs were established for each action during the first workshop, each with between one and four members. All sites completed both cycles of PDSA, although some actions were discontinued in cycle two due to the limited progress made or other changes happening outside of the study. Although 58 people took part, participation shifted over time due to new emerging stakeholders and also the challenges to some frontline nursing staff of being able to attend all meetings. Participation therefore varied from four people attending a workshop to 14 (see Table 4).

Implementation plans indicated seven main areas of work: 1) establishing a telehealth pathway; 2) improving patient assessment and review; 3) improving service delivery; 4) improving data sharing and access; 5) raising awareness of telehealth; 6) improving evaluation of telehealth; and 7) securing financial investment for telehealth. Table 5 indicates which sites worked on solutions to address these areas.

**Table 5:** Main activities for action research by site

Type of Activity	Site A	Site B	Site C	Site D
1. Establishing a telehealth pathway				
e.g. establishing clear referral criteria and process; considering options for effective discharge	✓	✓	✓	✓
2. Improving patient assessment and review				
e.g. reviewing current caseload of patients to establish goals for use and examine telehealth activity	✓	✓	✓	✓
3. Improving service delivery				
e.g. improving monitoring and triage of telehealth patients; establishing new roles for telehealth work	✓		✓	✓
4. Improving data sharing and access				
e.g. opening up data channels, improving electronic patient record systems	✓		✓	✓
5. Raising awareness of telehealth				
e.g. staff training and information sessions, championing, events to promote telehealth service	✓	✓	✓	✓
6. Improving evaluation of telehealth				
e.g. capturing telehealth activity on patient record system, identifying and agreeing outcomes to measure	✓	✓		
7. Securing financial investment for telehealth				
e.g. working with commissioners and industry, scoping out equipment needs for a future sustainable service		✓		✓

The following sections provides a summary of the work undertaken and the challenges encountered. Staff quotations from the focus groups are used to illustrate findings throughout.

### Establishing a telehealth pathway

All four sites chose to work on standardizing referral and discharge procedures, and clarify the duration of patient telehealth usage. This was to provide clarity about which patients were suitable for telehealth, and to address concerns that some patients no longer needed telehealth but had become dependent on the service. In the first cycle a period of further learning was initiated to help identify referral criteria, goals for use and discharge options. As part of this process, three sites developed step-down options (lower-level monitoring) to ensure that patients could be offered a staged approach to discharge. The second cycle provided an opportunity, in two sites, to pilot these options, which involved discharging some long-term users from the service. In one of these sites, this new practice was introduced following the action research.

*“We have offered some step down, so we’ve offered lean peripherals for patients and we’ve also offered telephone support calls. And it’s worked really well hasn’t it, for a lot we don’t need the peripherals because they’ve got their own.”* (Site A participant)

Two sites also piloted new electronic referral forms in cycle two. Feedback revealed difficulties with inter-operability between different electronic systems and restrictive data sharing protocols, which meant that some clinical teams could not access the new form. Participation of IT staff in the action research helped to identify potential solutions to address this, which were to be introduced following the end of the study.

### Improving patient assessment and review

All sites chose to improve the process for assessing and reviewing telehealth users to ensure efficient use of resources. This included collecting additional information from referring clinicians at assessment to identify goals for use and specify a date for review.

*“Now we do say to [patients] that it’s either for six weeks … so they know very well up front that it’s not there as a long term … on the referral form we’ve got now length of time and when we have put a couple of new patients on … we have put a review date on.”* (Site A participant)

To help determine how long patients should use telehealth for, all sites looked at the feasibility of introducing regular reviews. In the second workshop, participants identified a lack of clarity about what outcomes to measure and the limited availability of patient data as barriers to introducing this. Rather than abandon the activity, the researchers suggested completing an audit of current telehealth users to facilitate further learning, and worked with participants to design an audit template. The audit was completed in three sites during cycle two. Findings helped participants to identify which patients could be discharged from the service; what data to capture for reviewing patients; and how to refine existing referral and review criteria, for example, introducing a date for review of eligibility and options to share data across the patient’s care record.

### Improving service delivery

Three sites aimed to improve the processes for monitoring and triage of telehealth patients. Each site had previously completed some standardization, and therefore targeted specific elements of their service that were not working efficiently, or where practice was variable. Case study findings helped to identify which processes needing improvement.

*“I think the [case study] being an independent evaluation of how that kit was working, how it was used, was very useful because it was undermining a lot of the confidence in it, and if we’d actually just shouted about it, it would have been one provider shouting at another provider you know. Whereas it was an independent review of “is this working for patients?” … and it wasn’t.”* (Site C participant)

Additional analysis to identify common barriers and facilitators across the four sites in phase one also proved valuable, highlighting potential solutions which other sites had successfully introduced to improve their telehealth service. For example, two sites considered the feasibility of introducing a clinical role for triaging patients and providing telephone support, which in one site was initiated at the end of the study. Participants were partly motivated by learning that this had been successfully implemented in another study site, which having already created a telehealth nurse role, focused on developing protocols for the shared accountability of care between community nursing and telehealth staff. These sites also explored options for offering telehealth to patients outside the community nursing service, using the telehealth nurse role to provide the daily clinical advice and signposting to other services that would be required to support these patients.

Although some progress was made in each site, service improvements were inhibited by difficulties securing investment for new roles and equipment to support improved processes. Features associated with the current telehealth equipment also acted as a barrier, with limited flexibility and poor connectivity making it difficult to improve monitoring practices.

### Improving data sharing and access

Three sites worked on improving data sharing and access. Acknowledging that solutions to address the interoperability problems between monitoring software and electronic patient record systems were not readily available, participants considered other ways to reduce the additional workload associated with having to access two, and sometimes three, different systems. Other work focused on how to ensure that patient records were updated with monitoring information so that other clinicians involved in the care of a patient could access this information.

*“The first thing we’ve put on the [referral] template is “can you make sure your records are shared?”. We kind of built that in the template at the very beginning so hopefully it’ll prompt referral and the records being shared…we can’t make people press buttons, but it’s there as a prompt.”* (Site D participant)

Two sites worked with the company who had developed the electronic patient record system and local NHS IT departments, and were able to create new templates and links for accessing telehealth software and capturing telehealth activity. Attempts to open up data sharing between the different clinical teams involved in telehealth were hindered by interoperability problems and strict data protection protocols within local NHS Trusts, which as a wider organizational barrier would take time to address.

### Raising awareness of telehealth

All sites were keen to raise awareness of telehealth and ideas for action included training sessions for nursing teams who were reluctant to use telehealth; working more closely with new clinical commissioning groups; and hosting events to promote telehealth to other clinical providers. Because of competing priorities, this work was difficult to achieve and in two sites was discontinued after the first cycle. There were also mixed opinions about promoting a service that was, at the time, under threat due to the difficulties of securing investment for the next financial year. There were other concerns about the potential impact of generating interest in a service that had limited capacity and also needed improvement. Consequently, all sites agreed to delay this activity until an improved service could be implemented.

*“That would come with new kit … it’s very difficult to do much work with what we’ve got at the moment. We can go out and do training on what we’re using but it’s limited in its application and that makes it a barrier in itself.”* (Site D participant)

### Improving evaluation of telehealth

All sites agreed that better evaluation was required to understand more about telehealth outcomes. However, only two sites chose to work on this activity. Divided opinions about the rationale for investment in telehealth created uncertainties about which outcomes to measure and consequently, while participants agreed that this work was important, they could not agree on how to take it forward. Strict protocols for data sharing acted as an additional barrier to evaluation, as it limited the potential for accessing relevant data. External service providers faced further restrictions, which meant that they were not able to obtain basic patient data for evaluation. Some participants expressed frustration about not being able to prove the benefits of telehealth during the course of the study, as they believed that this was required to convince local decision-makers to re-invest in the service.

*“We can’t even tell whether the patients are benefiting in terms of the four objectives we were set … We have got no access to that [data] yet. So it’s kind of a catch 22 you know, we believe we are showing value and have some benefits for the patients.”* (Site C participant)

### Securing financial investment for telehealth

The short-term funding of telehealth was identified as a barrier to implementation. To secure future investment, participants focused on establishing relationships with technology providers and local decision-makers; scoping out the potential of new technologies that were available; and identifying the needs of users and clinicians that could be addressed with telehealth. Only one site was able to secure financial investment during the study timeframe, and in two sites there were real concerns about the future of telehealth.

*“We haven’t got much time to influence the commissioners you see and, for me it is about keeping the pressure on if you like because if we don’t and we don’t realise the potential of what we need to invest in there will be very little put into future tenders.”* (Site B participant)

The continuous work of telehealth champions helped to engage local decision-makers and convince them of the benefits of telehealth. Local evaluations and patient case studies also helped to reduce uncertainty; however, the lack of robust data and evidence of benefit acted as an enduring barrier to securing financial investment. Uncertainty about the future of telehealth also affected the views of some participants, who expressed disappointment that their efforts to improve telehealth may, ultimately, be fruitless. Other participants reflected positively on the action research process as a result of the shared learning and small improvements that had been achieved.

*“I think we are better placed to know what we want and therefore we are not happy just to accept oh this is what we’ve got, we actually have a vision now of how we are going to utilize it, we’ve learnt as we’ve gone along, it’s not been a complete waste of time…”* (Site A participant)

## Discussion

Action research and PDSA are promoted as useful tools for studying implementation and for improving service quality [27]. Although a recent evaluation of improvement projects identified key challenges to achieving goals for change and showed mixed results of impact [30], findings from this study demonstrate the prospective value of combining case study and action research methodologies to achieve planned incremental improvements and standardization to an existing telehealth service, a process that can otherwise be constrained by the temporal and practical barriers imposed by everyday practice [30]. Having said that, the potential for achieving change through action and cycles of improvement in this research was limited by the need throughout the study to determine the utility of telehealth and to prove evidence of benefit, which have both been identified elsewhere as key enablers for successful future integration of telehealth [2, 10, 15].

Ambiguity about telehealth and what it could achieve was prominent throughout the study. Uncertainty about how to implement sustainable telehealth led some participants to question the value of improving a service that was unlikely to be normalized due to the short-term funding arrangements and challenges to securing future investment, a barrier also identified in other studies of telehealth implementation [13, 17, 31]. Still, other participants were more pragmatic and believed that, to be able to expand telehealth in the future, understanding what works and why is an important first step to improve adoption and, ultimately, to normalize the practice of delivering care with new technologies into clinical routine. This dilemma within the study between having to demonstrate feasibility and effectiveness, or workability, and at the same time seek to understand the factors that lead to successful implementation, or integration [32], is a central concern for implementation research [32–34], and one that is captured by May [32], who draws attention to the social and political processes through which healthcare innovations are adopted, and subsequently normalised into practice.

May and Finch [33], through examining processes of implementation, including e-health initiatives [35], identified four generative mechanisms through which healthcare innovations are normalised: coherence; cognitive participation; collective action; and reflexive monitoring. In this research, there was evidence of all four mechanisms in operation across the study sites. This was accelerated in phase two of the project by employing principles of participatory action research and PDSA, which served to create a shared and dedicated regular space for stakeholders to work together in driving forward service improvements and engage in continuous sense-making, a cyclical and reflective process through which coherence can be achieved [36]. The role of researchers in the action research also helped to stimulate cognitive participation and reflexive monitoring, through employing facilitation skills as a mechanism to break down power structures between and within stakeholder groups and encourage collective planning and action. Additionally, drawing upon the case study findings and other research helped to stimulate the sense-making work associated with cognitive participation.

The underpinning PDSA approach in phase two helped to focus participants on making improvements, generating iterative cycles of collective action and reflexive monitoring. This shared practice of continuous sense-making also helped to shape plans for future expansion and evaluation of telehealth, and provided an opportunity to discuss uncertainties that remained. For example, which patients would benefit the most and how to determine the optimal duration for telehealth; issues that have yet to be addressed in studies of effectiveness. In fact, the mixed opinions and understanding of telehealth observed during both study phases, which are a barrier to frontline staff adoption [15], meant that achieving greater coherence was, in some sites, prioritised over action, and became an outcome in itself. Although wider enduring organisational and technological barriers impeded service delivery and improvement efforts, the importance of sense-making is identified in other recent studies examining implementation [19, 36].

The action research methodology proved fruitful in stimulating the social mechanisms associated with normalisation. However, the achievements accomplished during the study period would not have been possible without the continual work of telehealth champions, who helped to prioritize the research agenda and engage clinical teams. The important role local champions play in encouraging others to adopt new innovations is well recognized, and Wade et al., argue that they play ‘a critical role in the subsequent conversion of a project to an ongoing service’ ([17]: p688). Findings from the study reported here support this assertion; however, they also draw attention, as do Hendy and Barlow [37], to the complex system of stakeholder networks, infrastructures and processes that local champions must contend with, and the personal investment and passion that is required to initiate change. This raises questions about who should take responsibility for delivering such a complex service, and the training and support that may be required if frontline staff are expected to add these duties to their existing role.

The multiple stakeholder map and fragility of adoption processes raises an equally important question about who should act as the local lead for service improvement projects, and the implications this might have for engaging relevant stakeholder groups. In this study, despite the combined efforts of the research team and telehealth champions, certain key actors, including primary care providers and new clinical commissioners, were difficult to engage, without whom it proved difficult to embed change and make improvements or expansions that required additional finance. What’s more, although feedback was sought from users of telehealth about discharge options and new equipment, their exclusion as stakeholders in the action research raises questions about whom the service improvements were for, and whether or not they reflected the needs of end users. These points demonstrate the importance of engaging all stakeholders as equal partners if we are to maximize the potential for achieving change through action and ensure that plans for improvement meet the needs of all parties [38].

Difficulties measuring the impact of action research pose an additional limitation of using this method for service improvement and learning [25]. In this study, improvement was defined in various ways, depending on local acceptance and opinion of telehealth, and the implementation issues that emerged over time in each site. This made it difficult for participants to select outcomes to measure, and although the process of sense-making and the incremental improvements and standardization were reported to improve telehealth adoption, in reality the impact of these changes were difficult to quantify.

The non-linearity and inter-relatedness of adoption processes pose another challenge for research that attempts to generate knowledge about the factors that may enable, or alternatively, inhibit adoption of new innovations, or that focuses on implementation without paying attention to how this may influence, and be influenced by, adoption at the individual, organisational and national levels [10, 18]. The longitudinal nature of this research, which enabled processes of adoption to be studied in four sites over a two year period, further validates the need to examine implementation over time [1], which in this study increased understanding of the inter-relatedness of organisational, technological and clinical changes required to embed telehealth into a healthcare system that is constantly in flux; and the difficulties of driving forward such change when one or two key barriers cannot be overcome.

## Conclusions

Mainstreaming telehealth remains on the policy agenda, and despite the mixed evidence of effectiveness, solutions for embedding the use of remote care technologies continue to be pursued [39]. In this study, action research and service improvement methodology helped to accelerate key social mechanisms associated with the normalisation of new innovations, and allowed them to flow more freely across multiple stakeholder networks. However, while key improvements were achieved, uncertainty about the future of telehealth remained, and the appropriateness of employing these methods to embed complex innovations into routine practice is less certain, particularly when, for interventions like telehealth, consensus around evidence of clinical and cost effectiveness is yet to be established. Uncertainty about evidence of benefit was identified as an enduring barrier to mainstreaming telehealth, affecting adoption in practice and making it difficult to secure investment within organizations increasingly driven to prioritize cost savings over other, equally important, policy goals.

The study timing is also an important consideration, because of the type of telehealth applications being deployed and the significant organisational and technological changes within the English National Health Service during the period of research [40]. In the study sites, this resulted in many of the local decision-makers changing, existing networks being weakened or dismantled, and services and technologies being re-configured and modernised. These changes acted as a barrier to engaging relevant stakeholders in the action research, and limited the capacity of some participants to achieve change due to other emerging priorities. The study findings should therefore be interpreted temporally, and it could be that in a more static climate, service improvement projects are more successful; however, the interplay of factors that can affect adoption and implementation of new and complex innovations are likely to require an improvement approach that is embedded into service design, as opposed to a standalone project.

Like other research, this study draws attention to the limitations of applying strictly controlled trial conditions to examine the effects of telehealth [4, 31], which as a technology is still, to some extent, at an early stage of being developed and understood [41]. Although further developmental and feasibility research could help to determine why and how telehealth can be applied in particular settings, the complexity of technology-enabled care services means that evidence of cost effectiveness may on its own not provide the certainty that will encourage investment and normalisation [20, 41]. Increasingly, evaluations of complex interventions adopt a range of different approaches and methods to understand the processes and factors affecting implementation [27, 31, 42, 43]. However, for complex technologies that involve new stakeholders, networks and data not previously part of the healthcare environment, perhaps research now needs to turn its attention to identifying and testing new outcomes in order that future studies can apply more appropriate techniques for measuring the impact of telehealth, and to help inform the development of robust tools to evaluate local service provision.
